# Halide Perovskites: Thermal Transport and Prospects for Thermoelectricity

**DOI:** 10.1002/advs.201903389

**Published:** 2020-04-16

**Authors:** Md Azimul Haque, Seyoung Kee, Diego Rosas Villalva, Wee‐Liat Ong, Derya Baran

**Affiliations:** ^1^ KAUST Solar Center Physical Science and Engineering Division King Abdullah University of Science and Technology Thuwal 23955‐6900 Saudi Arabia; ^2^ ZJU‐UIUC Institute College of Energy Engineering Zhejiang University Hangzhou Zhejiang 310027 China; ^3^ State Key Laboratory of Clean Energy Utilization Zhejiang University Hangzhou Zhejiang 310027 China

**Keywords:** halide perovskites, hybrids, perovskites, phonons, thermal transport, thermoelectrics

## Abstract

The recent re‐emergence of halide perovskites has received escalating interest for optoelectronic applications. In addition to photovoltaics, the multifunctional nature of halide perovskites has led to diverse applications. The ultralow thermal conductivity coupled with decent mobility and charge carrier tunability led to the prediction of halide perovskites as a possible contender for future thermoelectrics. Herein, recent advances in thermal transport of halide perovskites and their potentials and challenges for thermoelectrics are reviewed. An overview of the phonon behavior in halide perovskites, as well as the compositional dependency is analyzed. Understanding thermal transport and knowing the thermal conductivity value is crucial for creating effective heat dissipation schemes and determining other thermal‐related properties like thermo‐optic coefficients, hot‐carrier cooling, and thermoelectric efficiency. Recent works on halide perovskite‐based thermoelectrics together with theoretical predictions for their future viability are highlighted. Also, progress on modulating halide perovskite‐based thermoelectric properties using light and chemical doping is discussed. Finally, strategies to overcome the limiting factors in halide perovskite thermoelectrics and their prospects are emphasized.

## Graphical abstract

Halide perovskites exhibit ultralow thermal conductivity and intriguing phononic behavior. This work elucidates the thermal transport behavior in halide perovskites and highlights their potentials and challenges for thermoelectric application.

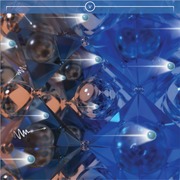

## 1 Introduction

Halide perovskites have emerged as a new class of semiconductor material with applications in a wide arena of optoelectronics. They have unique merits as photovoltaic materials exhibiting high absorption coefficient, low trap density, bandgap tunability, high photoluminescence (PL) quantum yield, and low‐cost solution processing.^[^ ^1^, ^2^, ^3^, ^4^, ^5^, ^6^, ^7^, ^8^ ^]^ In recent breakthroughs, halide perovskites challenge conventional wisdom in photovoltaic and LED research. Intriguing phenomena such as anomalous photovoltaic effect, phonon recycling, and optical cooling have been observed in halide perovskites.^[^ ^9^, ^10^, ^11^, ^12^ ^]^ For example, it was recently proposed that bright triplet excitons are behind the frequently observed high PL quantum yield in halide perovskites.^[^ ^13^ ^]^ Halide perovskites with the general chemical formula of ABX_3_, where A is an organic or inorganic cation, B a metal cation, and X a halide anion, present unique opportunities to realize versatile material properties through manipulating the cation and halide compositions. While alkali‐metal halide perovskites were synthesized in the early 1890s,^[^ ^14^ ^]^ the first report on 3D hybrid organic‐inorganic metal halide perovskites came about 80 years later in 1978.^[^ ^15^, ^16^ ^]^ Widespread research into their potential applications started decades later. The recent blooming research activities in 3D bulk halide perovskite photovoltaics was preceded by the successful application of 3D perovskites in the form of a nanocrystalline coating in dye‐sensitized solar cell.^[^ ^17^ ^]^ Whereas, their 2D layered counterparts, are being investigated since 1999 for their potential as electronic materials.^[^ ^18^ ^]^

In the initial few years, halide perovskite research community was solely focused on understanding the charge transport behavior of these materials. Recently, halide perovskites have started to gain interest for energy conversion devices beyond solar cells.^[^ ^19^ ^]^ Since the first report on ultralow thermal conductivity of CH_3_NH_3_PbI_3_,^[^ ^20^ ^]^ significant efforts were directed toward understanding its thermal transport, particularly on the phonon behaviors. Interestingly, halide perovskites are classified as having a “phonon glass, electron crystal”^[^ ^21^ ^]^ structure where charge transport is as efficient as in a good semiconductor while heat transport is hindered like in a glass.^[^ ^22^ ^]^ In addition, their room temperature Seebeck coefficient is generally found to be superior to other solution‐processed materials such as organics.^[^ ^23^, ^24^, ^25^, ^26^ ^]^ Their ultralow thermal conductivity coupled with decent carrier mobility and Seebeck coefficient led to the prediction of halide perovskites as a suitable candidate for future thermoelectrics.^[^ ^24^, ^27^ ^]^ While traditional inorganic thermoelectric materials such as Bi_2_Te_3_ have good thermoelectric performance, their high fabrication and material cost have motivated the quest for facile solution‐processed thermoelectric materials.^[^ ^23^, ^28^ ^]^ Optimization of solution‐processed thermoelectric materials such as organics, quantum dots, and hybrid composites are being continuously pursued to improve the cost‐performance matrix.^[^ ^29^, ^30^, ^31^ ^]^ If decent performance and good stability can be realized in emerging thermoelectric materials such as the halide perovskites, they can serve as a cost‐effective option from the viewpoint of simple fabrication and low material cost.

Though the understanding of thermal‐related behavior of halide perovskites is still in its infancy, significant progress is being made in terms of comprehending its phonon characteristics. The ultralow thermal conductivity of the halide perovskite solids mainly stems from their low phonon group velocities and short phonon lifetimes.^[^ ^32^ ^]^ In hybrid perovskites, the orientational disorder of the methylammonium (MA) cation, as well as the coupling of the vibrational modes of the inorganic cage and organic cation, governs the phonon characteristics and thermal transport. Interestingly, the dynamic disorder nature of the MA cation was already recognized more than three decades ago,^[^ ^33^, ^34^ ^]^ but its effect on phonon behavior and material properties was not studied until the recent re‐emergence of the halide perovskites.^[^ ^35^ ^]^

Although there have been several excellent reviews on halide perovskites,^[^ ^36^, ^37^, ^38^ ^]^ one focusing on perovskite thermal transport and thermoelectrics is still lacking. We will begin by discussing few fundamental concepts of thermal transport, thermoelectrics, and material property requirements for optimal thermoelectric performance. Since the stepping stone of any technology is the functional material itself, we briefly follow with reviewing the different types of existing perovskites (hybrid, inorganic, 3D and low dimensional). Then the observation of ultralow thermal conductivity, unique phonon transport behaviors, and compositional dependence will be discussed. Finally, we highlight the recent developments on halide perovskite‐based thermoelectrics and their challenges and limitations. We will conclude the review by discussing the opportunities offered by halide perovskites and propose several promising directions for the future development of halide perovskite‐based thermoelectrics as well as thermal management of such perovskite‐based devices.

## 2 Thermal Transport and Thermoelectrics

The thermal transport behavior of materials is generally quantified by the thermal conductivity (*κ*) parameter which, in turn, is governed by the Fourier's law.

(1) $$q^{\prime \prime }=-\kappa \nabla T$$

where ***q″*** is the heat flux vector and ∇*T* is the temperature gradient in three‐dimension.^[^ ^39^ ^]^ There is a strong correlation between a material's atomic structure and its thermal transport behavior.^[^ ^40^ ^]^ Both low and high *κ* materials are essential from an application perspective. High *κ* materials are useful for heat dissipation or thermal management applications while low *κ* materials are crucial for heat insulation and thermoelectrics. In general, low *κ* value is observed in electrically insulating amorphous solids and glasses, where the lower *κ* bound is often characterized by the Einstein or Cahill–Pohl limit.^[^ ^41^, ^42^, ^43^ ^]^ In contrast to amorphous materials, crystalline solids generally have higher *κ* values as well as higher carrier mobility that is important for electronic device applications. Considerable effort is undertaken to produce crystalline materials with low *κ* or to reduce the *κ* of such materials, especially for thermoelectric applications.^[^ ^44^ ^]^

Heat conduction in crystalline non‐insulating solids is primarily due to phonons that originate from the lattice vibrations in the materials and charge carriers (i.e., electrons and holes).^[^ ^45^, ^46^ ^]^ The electronic (*κ_e_*) and phononic or lattice (*κ_l_*) contribution to the total *κ* can be expressed as

(2) $$\kappa =\kappa_{e}+\kappa_{l}$$

The electronic component (*κ_e_*) is related to the electrical conductivity (*σ*) usually measured using a four‐point probe setup and estimated using the well‐established Wiedemann–Franz law.

(3) $$\kappa_{e}=L\sigma T$$

where *L* is the Lorentz number and *T* the temperature. The knowledge of the total *κ* and electrical conductivity of a material can, thus, be used to estimate *κ_l_*. There are a number of experimental techniques available to determine the total *κ* of a material such as the laser flash method,^[^ ^47^ ^]^ steady‐state method,^[^ ^48^ ^]^ time‐domain or frequency‐domain thermoreflectance,^[^ ^49^ ^]^ 3*ω* method,^[^ ^50^ ^]^ and so forth. Details of these individual methods can be found in the cited references as well as in other excellent reviews on thermal conductivity measurement techniques.^[^ ^51^ ^]^ On the other hand, *κ* can also be estimated theoretically. The simplest estimation invokes the phonon gas model where *κ_l_* can be approximated as

(4) $$\kappa_{l}=\left(1/3\right)C_{ph}\upsilon \Lambda$$

where *C_ph_* is the activated volumetric phonon heat capacity, *υ* is the average phonon group velocity, and *Λ* is the average phonon mean‐free path.^[^ ^52^ ^]^ The two former variables can be obtained using the harmonic lattice dynamics technique while the latter is an average from a spectrum of phonon mean‐free paths after various scattering mechanisms such as by other phonons, lattice defects, impurities, electrons, and interfaces.^[^ ^40^ ^]^ More accurate but computationally expensive methods exist to calculate *κ_e_* and *κ_l_*. Readers are encouraged to refer to recent reviews^[^ ^53^, ^54^ ^]^ for further details.

**Figure 1:**
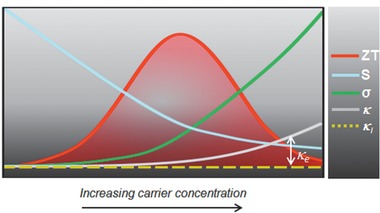
Schematic illustration of tradeoffs between various thermoelectric parameters.

Crystalline materials with intrinsic low *κ_l_* are particularly attractive for thermoelectric applications.^[^ ^45^ ^]^ In the past few decades, significant efforts have been directed to design materials with favorable thermoelectric properties. Thermoelectric efficiency of a device is evaluated by the thermoelectric figure of merit (*ZT*) and is defined as

(5) $$ZT=S^{2}\sigma T/\kappa$$

where *S*, *σ*, *κ*, and *T* are the Seebeck coefficient, electrical conductivity, thermal conductivity, and absolute temperature, respectively.^[^ ^44^ ^]^ For a high‐performance thermoelectric material (i.e., high *ZT*), it is imperative to have a high electrical conductivity, low thermal conductivity, and large Seebeck coefficient concurrently. The interdependency of these various parameters in *ZT* makes the material selection and optimization challenging (**Figure** **1**). One possible solution is to use heavily doped semiconductors with carrier concentrations between 10^19^–10^21^ cm^−3^ which can potentially balance the electrical conductivity and Seebeck conundrum to realize a high power factor (i.e., *S* ^2^ *σ*).^[^ ^46^ ^]^

## 3 Halide Perovskites

**Figure 2:**
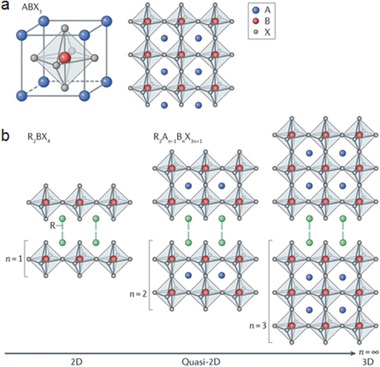
Structure of hybrid and inorganic perovskites. a) Illustration of the 3D hybrid perovskite structure ABX_3_, showing the corner‐sharing [BX_6_]^4−^ octahedra. A is an organic cation, B is a metal cation, and X is a halide. b) Illustration of the structures of low‐dimensional perovskites with different numbers of perovskite layers (*n*). The pure 2D perovskite (*n* = 1) has a R_2_BX_4_ structure, where R is a bulky organic cation. For *n > *1, the quasi‐2D perovskites arrange into a R_2_A*_n_*_−1_B*_n_*X_3_ *_n_*_+1_ structure. Reproduced with permission.^[^ ^61^ ^]^ Copyright 2018, Nature Publishing Group.

Most conventional semiconductors have rigid 3D structures. In hybrid perovskites, however, 3D to 2D transition can be triggered by replacing the A‐site cation with different organic cation groups (**Figure** **2**). Like organic semiconductors, hybrid perovskites allow the control of structural chemistry, tunability, and compositions by replacing the organic (A‐site) and metal cations (B‐site) as well as halide anions (X‐site). Combining MA or formamidinium (FA) organic cations with lead (Pb) or tin (Sn) metal cations creates the most frequently explored halide perovskites. To date, most of the highly efficient solar cells are MA/FA‐ and Pb‐based 3D hybrid halide perovskites. As Pb is toxic, efforts to replace it from halide perovskites have yielded several Pb‐free perovskites with promising light absorption property such as MASnI_3_ and CsSnI_3_.^[^ ^55^, ^56^ ^]^ However, these Sn‐based perovskites generally suffer from chemical stability issue, owing to the reactive nature of Sn in air. Other metal cations such as bismuth (Bi) and copper (Cu) have successfully formed perovskites in the form of (MA)_3_Bi_2_I_9_, Cs_3_Bi_2_I_9_, and (MA)_2_CuCl_2_Br_2_ as non‐toxic alternatives.^[^ ^57^, ^58^, ^59^ ^]^ Recently, many double perovskites based on mixing different metal cations into a single perovskite structure were reported with enhanced stability.^[^ ^60^ ^]^ Notwithstanding, their inferior light absorption, wide bandgap, and difficulty in processing have limited the use of non Pb‐ and Sn‐based perovskites.

Recently, low dimensional halide perovskites have attracted significant interest owing to their high PL quantum yield and enhanced stability compared to 3D perovskites. The dimensionality in halide perovskites is related to the corner‐sharing BX_6_ octahedra. In the case of a 3D perovskite, these octahedra share the corner atoms with one another, extending the structure into a 3D linked lattice. A 2D perovskite has a layered structure like in graphite, with each layer containing these linked octahedra while a 0D perovskite contains discrete octahedra that are isolated from one another.^[^ ^62^ ^]^ The dimension of the organic and inorganic motifs in these hybrids as well as the interface between them play a significant role in their underlying properties. With decreasing dimensionality, new physical phenomena start to emerge.^[^ ^63^, ^64^ ^]^ On the other hand, an all‐inorganic halide perovskite has its organic cations replaced by inorganic cations, with Cs^+^ in most cases.^[^ ^65^ ^]^ Interestingly, the 3D CsPbBr_3_ also has a 2D (CsPb_2_Br_5_) and a 0D (Cs_4_PbBr_6_) counterpart.^[^ ^66^ ^]^ Similarly, a dimensionality change in an inorganic halide perovskite can bring about unexpected ramifications to its electronic and thermal properties.^[^ ^67^, ^68^ ^]^

## 4 Thermal Transport in Halide Perovskites

Apart from thermoelectrics, understanding thermal transport in materials is critical for many vital processes. The thermal transport behavior plays a key role in determining the timescales of thermal energy transfer between the organic and inorganic moieties in hybrid perovskites.^[^ ^69^ ^]^ Also, the shorter phonon mean paths in halide perovskites can have ramifications on hot‐carrier cooling and recombination processes of solar cells.^[^ ^70^ ^]^ In this section, we will review the ultralow nature of *κ* in halide perovskites, their dependency on the chemical composition, and some of their intriguing phonon characteristics.

### 4.1 Ultralow Thermal Conductivity

**Figure 3:**
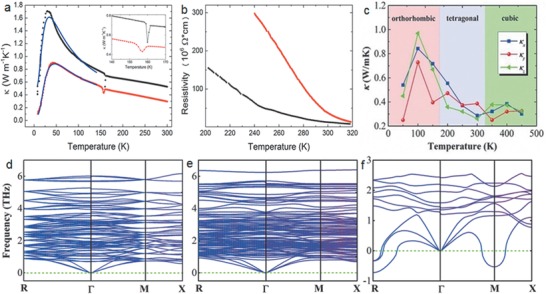
a) Temperature dependence of *κ* of single crystal (black) and polycrystal (red) MAPbI_3_ samples. Blue lines are obtained from the theoretical model. The inset shows the detail around the structural transition at 160 K. b) Resistivity as a function of temperature for single (black curve) and polycrystalline (red curve) samples. Reproduced with permission.^[^ ^20^ ^]^ Copyright 2014, American Chemical Society. c) Equilibrium molecular dynamics predicted anisotropic *κ* along various axes (*κ_x_*, *κ_y_*, and *κ_z_*) as a function of temperature. The phonon dispersion curves of d) orthorhombic, e) tetragonal, and f) cubic MAPbI_3_ in the low‐frequency domain. Reproduced with permission.^[^ ^32^ ^]^ Copyright 2016, Wiley‐VCH.

The first report on the *κ* of a hybrid perovskite,^[^ ^20^ ^]^ MAPbI_3_, came 5 years after the successful demonstration of a perovskite‐based solar cell in 2009.^[^ ^17^ ^]^ Pisoni et al. measured the temperature‐dependent *κ* of MAPbI_3_ and observed an ultralow value of 0.5 and 0.3 Wm^−1^ K^−1^ at room temperature for single‐crystal and polycrystal samples, respectively (**Figure** **3**a). The electrical resistivity values for the single‐crystal and polycrystal samples were in the order of 10^6^ ohms with the latter being more resistive due to constrained charge transport at the grain boundaries (Figure 3b). Similarities in the temperature dependence *κ* trend and comparable *κ* values for single‐crystal and polycrystal MAPbI_3_ samples indicates the intrinsic nature of the observed ultralow *κ*. Using the Wiedemann–Franz law, the electronic contribution (*κ_e_*) to the total *κ* was estimated to be negligible, indicating that heat is mainly transported by phonons. It was proposed that the low *κ* originates from the crystal structure of MAPbI_3_, with the slowly rotating CH_3_NH_3_^+^ cations within the unit cell suppressing the thermal transport. This pioneering work spurred interest in understanding the thermal transport mechanisms inside halide perovskites, resulting in a series of subsequent works to elucidate the phonon properties in these materials.

#### 4.1.1 Theoretical *κ*

In a theoretical work based on equilibrium molecular dynamics simulations, Lin et al. found that *κ* for all phases (cubic, tetragonal, orthorhombic) of MAPbI_3_ is below 1 Wm^−1^ K^−1^, with a room temperature value reaching as low as 0.31 Wm^−1^ K^−1^.^[^ ^32^ ^]^ The calculated *κ* values exhibited directional anisotropy at lower temperatures due to the structural anisotropy of the MAPbI_3_ crystal, with the orientation of the MA cation playing a crucial role (Figure 3c). In general, phonon properties directly determine the *κ* of semiconductors and insulators. The phonon dispersions for all three phases of MAPbI_3_ show overlapped phonon branches with dense optical branches starting at low frequencies (Figure 3d–f). Heat is mainly carried by the acoustic branches and low‐lying optical branches with non‐negligible group velocities. Enhanced phonon–phonon scatterings from these overlapped phonon branches lead to short phonon lifetimes (<100 ps) and mean‐free path (<10 nm). In addition, low phonon group velocities were predicted owing to the low elastic stiffness of MAPbI_3_. This phonon‐based theoretical study points to a very low *κ* in MAPbI_3_. Br‐ or Sn‐based hybrid perovskites exhibit higher *κ* in contrast to I‐ or Pb‐based hybrids due to their higher elastic moduli. The predicted ultralow *κ* of MAPbI_3_ in this work is in good agreement with the experimentally observed values by Pisoni et al.^[^ ^20^ ^]^ In another related work, Yang et al. calculated the *κ_l_* of MAPbI_3_ using equilibrium molecular dynamics simulations in the temperature range 160 to 400 K.^[^ ^71^ ^]^ A low *κ* value of 0.59 Wm^−1^ K^−1^ was obtained for the mid‐temperature tetragonal phase while a high *κ* value of 1.8 Wm^−1^ K^−1^ for the high‐temperature pseudocubic phase was predicted. Shorter lifetimes of the optical phonon branches, low group velocity of the acoustic phonons, and strong anharmonicity were found to be responsible for the low tetragonal phase *κ* value.

#### 4.1.2 Theoretical Study of Cation Dynamics on *κ*

To elucidate the role of cation dynamic disorder on thermal transport, Mattoni et al. using molecular dynamics based on ab‐initio derived potentials, revealed that the ultralow nature of *κ* in MAPbI_3_ is due to its low sound velocity.^[^ ^72^ ^]^ Suppressing the MA molecular rotation alone has negligible effect on the ultralow *κ*. Stopping all the internal vibrational modes of the MA molecule, however, doubled the *κ* value. This result contrasts with that by Yamashita et al. who developed a different set of ab‐initio based empirical potentials to evaluate the *κ* of MAPbI_3_.^[^ ^73^ ^]^ In this study, the rotational and translational motions of the MA cations interact with the inorganic Pb‐I octahedra to efficiently scatter the inorganic lattice vibration modes, thereby suppressing phonon transport. Results from first principle calculations from Hu et al. also agree that strong scattering between the rotational modes of the MA cation and the low‐frequency phonon modes of the inorganic lattice is the origin of the ultralow *κ* in MAPbI_3_.^[^ ^74^ ^]^ In addition, the MA^+^ clusters influence the inorganic lattice vibrations by interacting with the I^−^ ions but not with the shielded Pb^2+^ ions that are located in the octahedra center. More details on the effect of cation dynamics will be discussed in the next section.

#### 4.1.3 *κ* of Thin Halide Perovskite Films

The thermal transport of material in its bulk crystalline form generally differs from that in its nano‐size form due to enhanced scattering at the latter's material and grain boundaries.^[^ ^75^, ^76^ ^]^ Hybrid perovskite thin films also exhibited such characteristics.^[^ ^77^, ^78^ ^]^ Using the time‐domain thermoreflectance method, Luo et. al. compared the *κ* for neat spin‐coated perovskite films on silicon and films coated on a mesostructured Al_2_O_3_ scaffold supported on a glass substrate.^[^ ^77^ ^]^ For the neat films, *κ* decreases with increasing temperatures, exhibiting the typical behavior of bulk crystalline materials due to stronger Umklapp scattering at the higher temperatures. In the case of MAPbI_3_/Al_2_O_3_ nanocomposite films, *κ* first increases at low temperature and then stays nearly constant with temperature, showing an amorphous‐like trend. This plateau in *κ* is attributed to the amorphous nature of Al_2_O_3_ scaffold and the dense interfaces between Al_2_O_3_ and MAPbI_3_. Interestingly, an abrupt change in *κ* was observed for the neat film at the phase transition temperature, while only a small dip in *κ* was detected in MAPbI_3_/Al_2_O_3_. This abrupt change in *κ* observed in these neat films was attributed to the preferential orientation and changes in the thickness of their ferroelectric domains that affect phonon scattering at the domain boundaries. Similar dips in *κ* for mechanically pressed polycrystal and single‐crystal MAPbI_3_ samples measured using the steady‐state technique were previously reported. The temperature range of these dips also coincided with the orthorhombic‐tetragonal phase transition.^[^ ^20^ ^]^ A recent work observed a similar discontinuity of in‐plane *κ* at the orthorhombic‐tetragonal phase transition for MAPbI_3_ films. Density functional theory (DFT) calculations indicated a collapse in the phonon group velocity along the c‐axis of the tetragonal crystal resulting in the *κ* discontinuity.^[^ ^79^ ^]^

In addition to the traditional methods of measuring *κ*, new techniques have also been proposed to measure the thermal transport of halide perovskites, particularly for freestanding thin samples. Zhang et al. developed a micro‐PL spectroscopy technique to measure the *κ* of suspended single crystalline MAPbI_3_ platelets grown by chemical vapor deposition.^[^ ^80^ ^]^ A thickness‐independent room‐temperature *κ* of 0.14 Wm^−1^ K^−1^ was extracted. In another interesting work, a novel nanoscopic mapping technique based on scanning near‐field thermal microscope was developed to simultaneously map the thermal conductivity, thermal diffusivity, and volumetric heat capacity of all‐inorganic halide perovskite thin films.^[^ ^81^ ^]^ *κ* values of 0.43 and 0.33 Wm^−1^ K^−1^ were recorded for CsPbBr_3_ and CsPb_2_Br_5_ films, respectively.

#### 4.1.4 Anisotropy and In‐Plane Thermal Transport

More recent works have started to focus on the in‐plane and the anisotropic nature of the thermal transport in perovskite films. In one work, using a chip‐based 3*ω* technique, a *κ* value of 0.32 Wm^−1^ K^−1^ at room temperature along in‐plane direction was recorded for sequential vapor deposited MAPbI_3_ films.^[^ ^82^ ^]^ Using a similar technique, Fenwick et al. recorded an in‐plane *κ* of 0.31–0.59 Wm^−1^ K^−1^ at room temperature for MAPbI_3_ films with thicknesses varying from 65 to 100 nm.^[^ ^79^ ^]^ In another work, using a novel technique of light‐induced transient diffraction grating, spatial anisotropy of *κ* in vapor‐deposited hybrid perovskites was determined.^[^ ^83^ ^]^ For MAPbI_3_, a *κ* value of 0.19 Wm^−1^ K^−1^ along the in‐plane direction was recorded as compared to 0.28 Wm^−1^ K^−1^ for the cross‐plane direction. Hybrid perovskites of pure I, Br, Cl as well as their mixed compositions (IBr, ClBr) exhibited spatial anisotropy. For MAPbCl_3_, a *κ* value of 0.5 Wm^−1^ K^−1^ along the in‐plane direction was measured in contrast to the 1.1 Wm^−1^ K^−1^ measured for the cross‐plane direction. The spatial anisotropy was attributed to the thermal transport characteristics of the crystallite grains. Non‐spherical grains can lead to unequal scattering rates of acoustic phonons at the grain boundaries along the in‐plane and cross‐plane directions. Phonons traveling along the cross‐plane direction might experience less scattering compared to the in‐plane direction due to lesser grain boundaries in the former. Furthermore, orientational ordering within the layer can also influence the sound of speed leading to the observed spatial anisotropy. Such spatial anisotropy of *κ* can play a crucial role for perovskite optoelectronic devices such as solar cells and LEDs.

**Figure 4:**
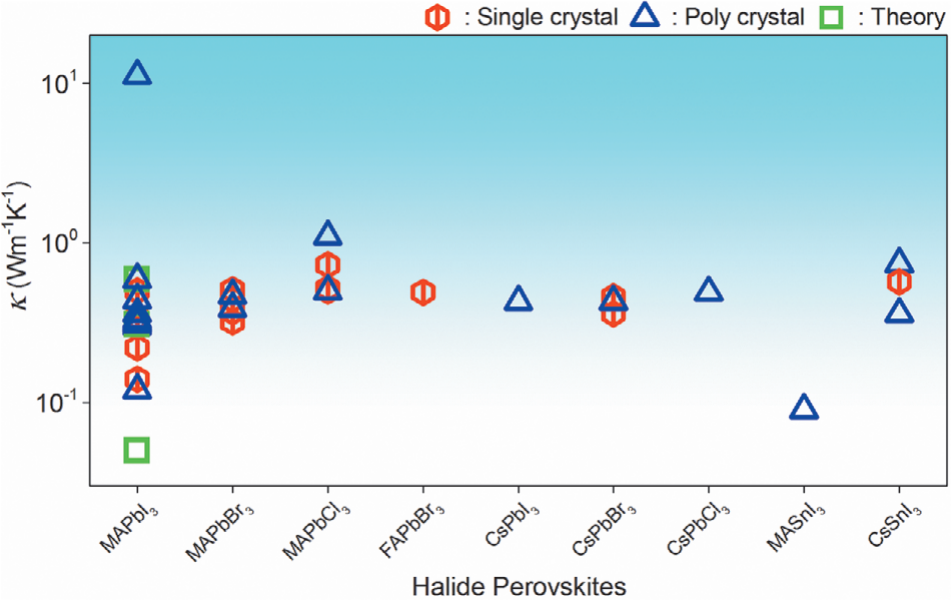
Room temperature experimental and theoretical *κ* for halide perovskites.^[^ ^20^, ^32^, ^70^, ^71^, ^78^, ^79^, ^80^, ^81^, ^82^, ^84^, ^85^, ^86^, ^87^, ^88^, ^89^, ^90^, ^91^, ^92^, ^93^, ^94^ ^]^

Though there are some discrepancies in the *κ* value for MAPbI_3_ from existing literature, almost all of them agree with its ultralow nature. In an isolated case, a high *κ* of 11.2 Wm^−1^ K^−1^ was measured for densely packed MAPbI_3_ films using the time‐domain thermal reflectance technique.^[^ ^78^ ^]^ The reason behind this high value is yet to be known. **Figure** **4** shows a summary of the literature room temperature in‐plane and cross‐plane *κ* for the halide perovskites in different forms (i.e., single crystalline, polycrystalline pellets, and thin films). The *κ* value spans over a wide range for MAPbI_3_. Compared to MAPbI_3_, reports on the thermal transport of other halide perovskites are fewer. From large spread of *κ* values for some of these perovskites as shown in Figure 4, indicates that more efforts are needed to understand the thermal transport in these materials. *κ* measurements are generally used to study the thermal transport properties but can be useful to infer other properties. For instance, Burda et al. employed *κ* measurements to track the degradation of MAPbI_3_.^[^ ^95^ ^]^ It was found that mild atmospheric aging leads to a thermal percolation threshold where the *κ* increases due to the formation of a small quantity of PbI_2_ in the MAPbI_3_ sample. These authors proposed the possibility of nanostructuring perovskite grains by including the trap passivation effect of PbI_2_ and its high *κ* to improve the heat dissipation of perovskite‐based devices.

### 4.2 Compositional Dependency of Thermal Conductivity

The crystal structure and chemical composition play a crucial role in determining the charge as well as thermal transport of halide perovskites. Initial experimental and theoretical works predict a significant role played by the A‐site organic cation and its interactions with the inorganic cage in determining the thermal transport in hybrid perovskites.^[^ ^20^, ^32^, ^74^ ^]^ In addition, the effect of changing the B‐site metal and X‐site halide on thermal transport were also recently investigated, broadening our understanding in this class of materials.^[^ ^86^, ^89^ ^]^

#### 4.2.1 Effect of A‐Site Cation

**Figure 5:**
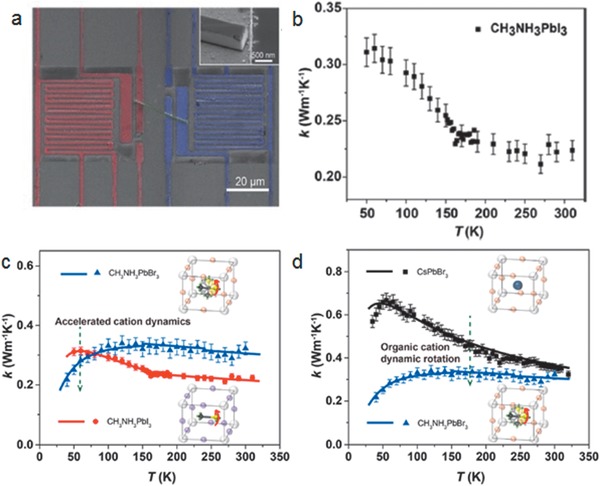
Thermal measurement of perovskite nanowires. a) False‐color SEM image of typical microbridge device with a perovskite nanowire bridging between two suspended pads. The heat flow is defined from heating membrane (red) to sensing membrane (blue) through the nanowire (green). (Inset) High‐magnified SEM image of typical measured nanowire. b) *κ* of corresponding MAPbI_3_ nanowire. The dip around 160 K coincides with the orthorhombic‐to‐tetragonal phase transition. *κ* of CsPbBr_3_, MAPbBr_3_, and MAPbI_3_ nanowires. c) *κ* comparison between MAPbBr_3_ and MAPbI_3_ reveals the role of accelerated cation dynamics in suppressing *κ* (green arrow in c) at low temperature. d) *κ* comparison between CsPbBr_3_ and MAPbBr_3_ reveals the role of cation dynamic in suppressing *κ* (green arrow in d). Insets in (c,d) are the simplified schematic diagrams of MAPbI_3_, MAPbBr_3_, and CsPbBr_3_ unit cell with indication of cation dynamic motion in hybrid perovskite (red arrow in the unit cell). Scattered data points are the measurement results and the solid lines are the model fitting curves. Reproduced with permission.^[^ ^85^ ^]^ Copyright 2018, American Chemical Society.

To gain further insights on how A‐site cation and halide anions affect thermal transport, Zhu et al. experimentally investigated three (MAPbI_3_, MAPbBr_3_, and CsPbBr_3_) prototypical halide perovskites in the form of a nanowire.^[^ ^85^ ^]^ Thermal measurements were accomplished using the microbridge technique resulting in an ultralow *κ* of 0.22 Wm^−1^ K^−1^ at 300 K for MAPbI_3_ nanowire (**Figure** **5**). This value is lower than previously reported values for single‐crystal and polycrystalline samples and can be attributed to the crystal orientation and smaller cross‐section area of the nanowires. For the temperature regime where both MAPbI_3_ and MAPbBr_3_ are in their orthorhombic phase, the *κ* for MAPbBr_3_ crosses over and becomes lower than that of MAPbI_3_. The larger lattice spacing with smaller halide atom size allows for more dynamic disorder of the MA cation. For the all‐inorganic CsPbBr_3_ nanowire, which maintains its orthorhombic phase in the whole measurement temperature range, a low value of 0.36 Wm^−1^ K^−1^ was recorded at room temperature. Interestingly, *κ* for MAPbBr_3_ is lower than CsPbBr_3_ irrespective of the phase, suggesting that the organic cation can be more effective in suppressing thermal transport, possibly due to the molecular dynamic disorder.

On the other hand, Dyck et al. compared the *κ* of MAPbI_3_ and CsPbI_3_ pellets to deduce the effect of MA cation on phonon scattering.^[^ ^92^ ^]^ The difference between the *κ* values for MAPbI_3_ and CsPbI_3_ is large at low temperatures but minimal at room temperature. It was proposed that since both MAPbI_3_ and CsPbI_3_ exhibit ultralow *κ* at room temperature, the dominant mechanism of phonon scattering at room temperature is Umklapp scattering from the Pb‐I cage. Resonant phonon scattering associated with the MA cation suppresses *κ* at low temperature, but its relevance at room temperature is relatively small. Malen et al. through measuring and analyzing the *κ* of several different halide perovskites, also found a common average acoustic phonon mean‐free path at room temperature for hybrid and inorganic halide perovskites, suggesting that the resultant effect of phonon scatterings in these different materials might be similar.^[^ ^86^ ^]^ In a recent work, different concentration of MAI in sequential vapor‐deposited MAPbI_3_ did not produce any significant changes in the *κ* value between 293–375 K, pointing to the dominant contribution from Pb‐I cage.^[^ ^82^ ^]^ Further evidence from neutron spectroscopy showed a similar range of phonon lifetimes for an all‐inorganic CsPbBr_3_ and a hybrid perovskite MAPbCl_3._ The disordered nature of the A‐site cation is, perhaps, universal in halide perovskites not just confined in hybrid perovskites.^[^ ^96^ ^]^ The necessity of an organic molecular cation to achieve an ultralow thermal conductivity is, however, debatable with most experimental evidences challenging simulation results. Experimental samples are typically less ideal than simulation models, a condition that can create such discrepancies. On the other hand, experimental measurements only yield an overall *κ* value that does not explicitly and readily separate the contributions of various energy carriers to *κ*. Highly efficient phonon scatterings can, perhaps, give rise to non‐phononic carriers that can also contribute to thermal transport, a postulation that should be further explored.^[^ ^97^ ^]^

#### 4.2.2 Effect of Halide

**Figure 6:**
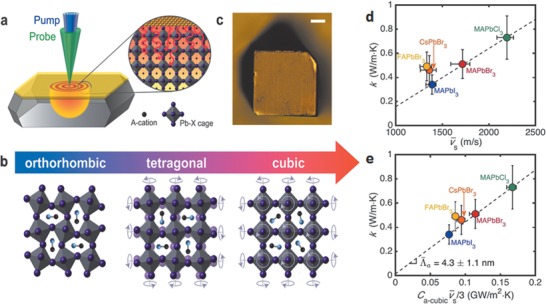
a) Schematic of the FDTR measurement on an Au‐coated perovskite single crystal. A pump laser (blue) heats the crystal while a probe laser (green) measures the temperature‐dependent reflectance of Au to determine the *κ* of the crystal. b) Common phase transitions of the perovskite lattice, shown for MAPbX_3_. In the tetragonal and cubic phases, the MA cations are under constant motion within the lattice and are therefore shown in random orientations. The purple arrows in these phases illustrate some of the many possible octahedron motions of the system under dynamic disorder. c) Optical microscope image of a CsPbBr_3_ crystal with an Au transducer layer evaporated on top. Scale bar is 50 µm. d) *κ* as a function of sound speed (*v̅_s_*). e) *κ* as a function of C_a‑cubic_ *v̅_s_*/3, where C_a‑cubic_ is the volumetric heat capacity of acoustic phonons based on a cubic unit cell. Reproduced with permission.^[^ ^86^ ^]^ Copyright 2017, American Chemical Society.

Most experimental thermal transport studies of halide perovskites use single crystals owing to the facile synthesis of large size crystals. In addition, single crystals offer high quality and grain boundary‐free samples. Feng et al. measured the *κ* values for three different halide variants of single‐crystal hybrid perovskites using the laser flash method.^[^ ^84^ ^]^ At room temperature, the obtained *κ* values were 0.30, 0.37, and 0.52 Wm^−1^ K^−1^ for MAPbI_3,_ MAPbBr_3_, and MAPbCl_3_, respectively. Also, the thermal expansion for the crystals measured by thermomechanical analysis exhibited ultrahigh values. The linear thermal expansion coefficient recorded for MAPbI_3_ in its tetragonal phase along [100] direction was as high as 57.8 × 10^−6^ K^−1^ with an even higher value of 263.5 × 10^−6^ K^−1^ around the tetragonal to cubic phase transition temperature. Such high thermal expansion may cause significant stability issues in real functional devices where temperature fluctuations can be common. From measuring a series of halide perovskites MAPbX_3_ (X = I, Br, Cl), FAPbBr_3_, and CsPbBr_3_ (**Figure** **6**), Malen et al. proposed that the *κ* of halide perovskites scales with the sound speed at the room temperature.^[^ ^86^ ^]^ These measurements revealed that the average mean free paths of the phonons are similar irrespective of the composition. In addition, ≈70% of the room temperature *κ* is a direct consequence of phonons with mean free paths shorter than 100 nm. The difference in the measured *κ* can, thus, be attributed to the difference in the sound velocity and heat capacity of the halide perovskites which directly affect the lattice thermal conductivity as seen from Equation (4). In another work, Heiderhoff et al. measured the *κ* for single crystals and films of MAPbX_3_ (X = I, Br, Cl) using scanning near‐field thermal microscopy to understand the effect of sample thickness, morphology, and composition.^[^ ^87^ ^]^ At room temperature, the obtained *κ* values were 0.34, 0.44, and 0.50 Wm^−1^ K^−1^ for MAPbI_3,_ MAPbBr_3_, and MAPbCl_3_, respectively. Similar values were obtained for the corresponding hot‐pressed thin films indicating the intrinsic ultralow nature of *κ*. There was a sharp increase in the *κ* value of MAPbI_3_ reaching 1.1 Wm^−1^ K^−1^ after the cubic phase transition temperature (55 °C). While this is consistent with a theoretical prediction of higher *κ* in the pseudocubic phase,^[^ ^71^ ^]^ this result seems to contradict a recent work that reported significant softening of phonons with strong acoustic damping in the cubic phase as well as near the cubic‐tetragonal phase transition.^[^ ^98^ ^]^ Such a behavior can lead to a reduced *κ*. The nature of the measured abrupt change in *κ* around the phase transition temperature will require further in‐depth study to pinpoint the origin of this contradiction which, perhaps, arises from differences in the quality or/and directionality of the experimental and simulated sample.

#### 4.2.3 Effect of B‐Site Metal

Only a few experimental and theoretical works reported the effects on thermal transport through changing the B‐site metal. The experimentally‐measured *κ* of MASnI_3_ shows a very low room temperature value below 0.09 Wm^−1^ K^−1^.^[^ ^89^ ^]^ This result contradicts the result of equilibrium molecular dynamics simulations where it was predicted that MASnI_3_ will have higher *κ* than MAPbI_3_ due to the higher elastic moduli of the former.^[^ ^32^ ^]^ Interestingly, first principle calculations on double perovskites Cs_2_AgBiCl_6_ and Cs_2_AgBiBr_6_ also yielded an extremely low *κ_l_* of 0.078 and 0.065 Wm^−1^ K^−1^ at 300 K, respectively.^[^ ^99^ ^]^ However, the total *κ* is very high (at 50–70 Wm^−1^ K^−1^ at 300 K) for these double perovskites due to their high electronic contribution. Such a high total predicted *κ* value indicates these compounds will have exceptionally high electrical conductivity. But a recent experimental work reported that single crystalline Cs_2_AgBiBr_6_ are very resistive in nature.^[^ ^100^ ^]^ The nature of this large discrepancy is unknown and requires further computational and experimental investigations to clarify. In another work, the computed *κ_l_* for the double halide perovskite Cs_2_InAgCl_6_ was found to be 0.2 Wm^−1^ K^−1^ at 300 K, due to its high intrinsic anharmonicity and low group velocity.^[^ ^101^ ^]^ With only a few thermal conductivity studies looking at the effect of changing the B‐site metal, more work will be needed to elucidate its influence.

### 4.3 Phonon Behavior

The characteristics of phonons are crucial for understanding not only the thermal transport of functional materials but also charge transport in the form of electron‐phonon interactions in polar semiconductors such as halide perovskites. Also, phononic behavior can be correlated with photophysical properties.^[^ ^102^, ^103^ ^]^ Several experimental and theoretical works have already established that orientational disorder and coupling exist between the vibrational modes of the inorganic cage and organic cations in hybrid perovskites.^[^ ^104^, ^105^ ^]^ Furthermore, phonon–phonon scattering in halide perovskites play a critical role in determining their thermal conductivity.^[^ ^72^, ^106^ ^]^ Open questions, however, remain for the phonons’ dependency on temperature and composition. In addition, the nature of the dominant heat carriers in such a material system can be further elucidated. In this section, some representative works on the phonon behaviors in halide perovskites and its ramifications are discussed.

#### 4.3.1 Non‐Phonon Framework

**Figure 7:**
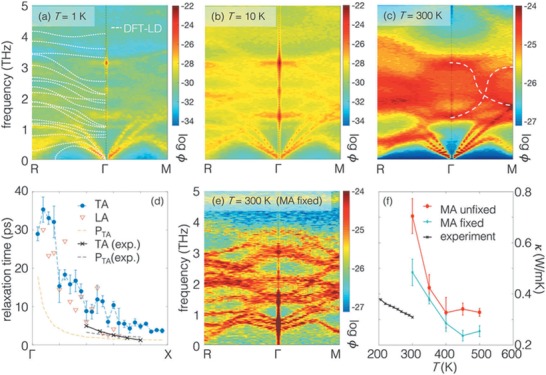
a–c) Spectral energy density of dynamical modes in MAPbI_3_ at different temperatures from ab initio molecular dynamics. The dotted white line for *T* = 1 K shows the lattice dynamics result for comparison. The band line‐widths broaden as temperature increases. At *T* = 300 K, the vibrational modes between 3–4 THz that are well‐defined at low temperatures are now heavily blurred. d) The modal relaxation time extracted from SED compared to recent experimental measurements. The relaxation time is close to the wave period for each thermal carrier, suggesting the conventional phonon picture is not an adequate description of the carriers. e) Freezing the rotations of the MA molecules recovers the well‐defined lattice dynamical modes, which suggests that interactions between the inorganic framework and sublattice MA orientational disorder are responsible for the loss of wave nature in (c). f) *κ* calculated via Green–Kubo formalism using classical molecular dynamics, with MA fixed and unfixed, compared with experiments. Reproduced with permission.^[^ ^97^ ^]^ Copyright 2018, Royal Society of Chemistry.

Phonons naturally arise from the periodic nature of a crystal.^[^ ^107^ ^]^ Although MAPbI_3_ has a crystalline structure, the validity of using the phonon picture to describe thermal transport in this material is challenged by the dynamic disorder of the MA molecules and the PbI_6_ lattice. A recent ab initio molecular dynamics coupled with spectral energy density analysis by Ertekin et al., however, offers insights into this problem.^[^ ^97^ ^]^ These hybrid perovskites are found to contain the glass‐crystal duality, where both phononic and non‐phononic (i.e., propagons, diffusons, and locons as described in the Allen Feldman theory^[^ ^108^ ^]^) elements are important for thermal transport. (**Figure** **7**). According to this study, non‐phononic carriers are created from the intense and coupled scattering of optical phonons that interferes and destroys their wave nature. These enhanced scattering events arise from the strong anharmonicity in this material that has origin from the resonant bonding within the inorganic lattice as well as the lattice coupling with the MA cations through their polarizability, dipole moment, and symmetry breaking effect. The scattering rate of the optical phonons are found to be orders of magnitude larger than in inorganic crystals agreeing with earlier reports.^[^ ^70^ ^]^ Similar to glassy materials, diffusons are the dominant thermal transport contributors in MAPbI_3_. Although propagons (resembling typical phonons) and locons (highly localized on A‐sites) also exist, they have negligible contribution to the overall *κ*. In contrast, these A‐sites are found as scattering sites for phonons under the phonon gas model and can, thus, affect the overall thermal transport.

#### 4.3.2 Phonon Framework

Inelastic neutron and X‐ray scattering are capable of generating experimental phonon dispersions^[^ ^106^, ^109^, ^110^, ^111^ ^]^ while terahertz, Raman, and infrared vibrational spectroscopy^[^ ^104^, ^112^, ^113^ ^]^ are other common techniques employed to experimentally study phonon modes. Early studies revealed the strong coupling between the organic cations and inorganic lattice shapes the vibrational spectra in these materials,^[^ ^109^, ^114^ ^]^ an observation that concurs with many theoretical studies.^[^ ^74^, ^105^, ^115^ ^]^ Recently, Toney et al. experimentally measured the acoustic phonon lifetimes in fully deuterated single crystals of MAPbI_3_ using high‐resolution inelastic neutron scattering^[^ ^106^ ^]^ and observed extremely short phonon lifetimes on the order of picoseconds. Using lattice dynamics calculations, they proposed that such short lifetimes originate from the strong three‐phonon interactions as well as the high density of low energy optical modes linked to the vibration modes of the organic cations. Another related work, using the high‐energy resolution inelastic X‐ray scattering technique on tetragonal phase MAPbI_3_, showed the presence of ultrasoft acoustic modes with low group velocities.^[^ ^110^ ^]^ These low group velocities together with short phonon lifetimes arising from strong ultrasoft acoustic‐low energy optical phonon scatterings are postulated to be responsible for the measured ultralow *κ*.

A temperature‐dependent study of the Pb‐I phonon modes in MAPbI_3_ (from 0.5 to 3 THz) revealed a splitting of two optical phonon modes into four when MAPbI_3_ cools and phase‐changes from a tetragonal to an orthorhombic structure.^[^ ^112^ ^]^ Electron mobility models utilizing the frequencies of the resulting four optical modes yielded reasonable agreement with experimental mobility measurements. This agreement suggests a possible correlation between the low‐energy optical phonon modes and the electronic transport properties in this material. Kanemitsu et al. observed a similar splitting of two phonons into four in MAPbX_3_ (where X = I, Br, and Cl) at the tetragonal‐to‐orthorhombic transition.^[^ ^116^ ^]^ From their experimental results, they suggested that the coupling of MA cations with the longitudinal optical (LO) modes of inorganic lead halide cage is responsible for the mode splitting at low temperatures and the damping of LO phonon modes at high temperatures. This coupling of the MA cations to the inorganic cage is facilitated through the hydrogen bond with the halide ion.^[^ ^113^ ^]^ In addition, by studying the evolution of phonon modes with temperatures, several studies concluded the coexistence of the orthogonal/tetragonal^[^ ^112^ ^]^ and tetragonal/cubic^[^ ^111^, ^117^ ^]^ phases across the respective transition temperatures. The coexistence of two different phases at a same temperature complicates the thermal transport picture.

While most of the thermal transport studies have focused on 3D halide perovskites, the thermal transport study of lower dimension perovskites such as 2D Ruddlesden–Popper hybrid perovskites and 0D perovskites have started to gain traction. A recent work demonstrated significant reduction in group velocity and propagation length of the coherent phonons cross the van der Waals bonded 2D perovskite layers that are terminated with organic molecules.^[^ ^118^ ^]^ Using a coarse‐grained bead‐spring model, the large acoustic mismatch between the organic and inorganic layers was found to suppress the acoustic phonon transport in these 2D perovskites. Compared to 3D perovskites, lower *κ* is observed in the case of 0D perovskites such as (MA)_3_Bi_2_I_9_ and Cs_4_PbCl_6_.^[^ ^93^, ^94^, ^119^ ^]^ Discrete octahedra structure with low group velocities, reduced phonon lifetimes, and the localization of vibrational energy leads to the ultralow *κ* in 0D perovskites.^[^ ^64^, ^93^ ^]^

In summary, works on the thermal transport of halide perovskites underscore the importance of chemical composition and structural phase to the phonon modes. The interactions between these modes ultimately regulate a perovskite's thermal and electronic properties. The presence of soft phonon modes and dense low‐lying optical modes with non‐negligible group velocities in these materials may have severe influences on their electron‐phonon coupling behavior. More work will be needed to fully clarify the nature and relative contributions of thermal carriers in this family of material at various temperatures.

## 5 Halide Perovskites Based Thermoelectrics

Compared to thermal transport studies, investigations into the thermoelectric performance of halide perovskites are still in its infancy. While halide perovskites exhibit ultralow *κ* and high Seebeck coefficient, the power factor and *ZT* are quite low in general due to their electrically resistive nature. The present thermoelectric performance of halide perovskites is significantly lower than the state‐of‐the‐art inorganic thermoelectric materials such as Bi_2_Te_3_, Cu_2_S, SnSe, lead chalcogenides where the *ZT* value is generally higher than 1.^[^ ^120^, ^121^, ^122^, ^123^ ^]^ In the case of organics and hybrids, their best *ZT* values lie between 0.4–0.6.^[^ ^124^, ^125^, ^126^ ^]^ The current highest *ZT* for halide perovskites is in the range ≈0.1.^[^ ^88^, ^90^, ^127^ ^]^ Since halide perovskite thermoelectrics is an emerging field, it will take extensive efforts to improve their performance to compete with traditional thermoelectric materials similar to the decades of research and optimization required for traditional thermoelectric materials. Research in perovskite thermoelectrics is nascent and accelerated only in the last few years. Comparing with organic thermoelectrics, its performance is on par with some existing organic and hybrid counterparts.^[^ ^128^ ^]^ The major advantage of perovskite thermoelectrics is its cost‐effectiveness, putting them as a viable option to explore. Also, the intrinsic ultralow *κ* and decent Seebeck coefficient at room temperature makes them lucrative for further research. Recent theoretical work predicts even lower *κ* can be achieved using perovskite superlattices.^[^ ^129^ ^]^ With improved stability and decent performance, perovskites can be used for low grade heat recovery and for electronic devices requiring low power to begin with.

**Figure 8:**
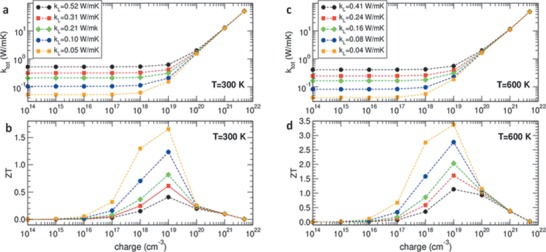
Total *κ* = *κ_el_* + *κ_l_* (a,c) and the dimensional figure of merit *ZT* (b,d) versus charge concentration for electron‐doped MAPbI_3_, calculated at two different temperatures; *κ_l_* is extrapolated from the experimental data published elsewhere. Black dots and red squares correspond to the experimental *κ_l_* values for single crystal MAPbI_3_ and poly crystal MAPbI_3_, respectively; the other symbols are for rescaled *κ_l_* values (indicated in the legend), mimicking eventual *κ* suppression occurring in 2D wells of thickness *L* = 40 nm (green diamonds), 20 nm (blue circles), and 10 nm (orange squares). Reproduced with permission.^[^ ^131^ ^]^ Copyright 2014, American Chemical Society.

In this section, we review several representative theoretical and experimental works on the thermoelectric performance of halide perovskites. In one of the early reports, Kanatzidis et al. measured the Seebeck coefficient of a series of halide perovskites single crystals and pellets, observing a dependence of the type of major carrier on the growth conditions.^[^ ^24^ ^]^ In particular, MAPbI_3_ and FAPbI_3_ demonstrated giant Seebeck coefficients around 10^5^ and 10^6^ µV K^−1^ at room temperature, respectively. These values are much higher than 10^2^ µV K^−1^ for the state‐of‐the‐art Bi_2_Te_3_ thermoelectric material.^[^ ^130^ ^]^ In one theoretical work, Mattoni et al. analyzed the thermoelectric performance of MAPbI_3_ as a function of temperature and carrier concentration.^[^ ^131^ ^]^ Electron doping appeared to be more efficient than hole doping in achieving higher thermoelectric performance due to the smaller band isotropy of the holes. *κ_l_* dominates the total *κ* for a carrier concentration from 10^14^ to 10^18^ cm^−3^. Above this upper limit, the contribution from *κ_el_* becomes significant. The *ZT* value peaks at a carrier concentration around 10^19^ cm^−3^ for temperatures at 300 and 600 K. High *ZT* values greater than 1 and 2 were obtained at 300 and 600 K, respectively by considering a hypothetical 2D well (**Figure** **8**).

**Figure 9:**
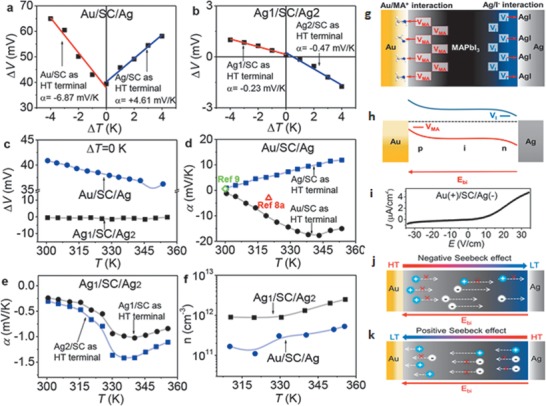
Anomalous Seebeck effect. Potential difference as a function of temperature difference for a) Au/SC/Ag sample and b) Ag1/SC/Ag2 sample at 313 K. c) Temperature‐dependent potential difference when Δ*T* = 0 K. d) Temperature‐dependent Seebeck coefficients for Au/SC/Ag sample when using Au/SC and Ag/SC surfaces as HT terminal, respectively. e) Temperature‐dependent Seebeck coefficients for Ag1/SC/Ag2 sample when using Ag1/SC and Ag2/SC surfaces as HT terminal, respectively. f) Temperature‐dependent carrier density. Schematic diagram of g) metal/ion interactions at metal/MAPbI_3_ interfaces, h) metal/ion interactions induced p‐i‐n junction across a MAPbI_3_ single crystal. i) Dark current density‐electrical field (J‐E) characteristics of Au/SC/Ag sample. j) Diagram of negative Seebeck effect due to electron‐dominant diffusion. k) Diagram of positive Seebeck effect due to hole‐dominant diffusion assisted by built‐in field caused by the p‐i‐n junction. Reproduced with permission.^[^ ^132^ ^]^ Copyright 2014, American Chemical Society.

Modulation of the sign of the Seebeck coefficient was demonstrated by Hu et al. on MAPbI_3_ single crystals by using the ion–electrode interactions at the crystal surface.^[^ ^132^ ^]^ The crystals used in the study were slightly n‐doped, as revealed by the Hall measurements. Au and Ag contacts were deposited as electrodes on the opposite faces of the crystal for Seebeck measurements. A negative Seebeck coefficient was recorded when the Au contact served as a high‐temperature terminal in contrast to a positive Seebeck coefficient when the Ag contact was used. For symmetric Ag electrodes on both faces of the crystal, a negative Seebeck effect was observed (**Figure** **9**). High positive and negative Seebeck values in the mV K^−1^ range were recorded. The origin of the negative and positive Seebeck coefficient was proposed to be an ion–electrode interaction where MA^+^ ions migrate toward the Au electrode and I^−^ migrates to the Ag electrode. This migration results in the creation of MA^+^ vacancies and I^−^ vacancies near the Au and Ag electrodes, respectively and the formation of a p‐doped region at the Au/crystal interface and n‐doped region near Ag/crystal interface, forming a p‐i‐n junction across the crystal. The internal field generated by the p‐i‐n junction regulates the dominant carrier (electrons or holes) flow depending on the electrode (Au or Ag) used as high‐temperature terminal. In another work, anisotropic Seebeck coefficients were recorded for a MAPbBr_3_ crystal in the in‐plane and cross‐plane directions.^[^ ^133^ ^]^ Positive and larger in magnitude Seebeck values were obtained for the cross‐plane direction in contrast to the negative values for the in‐plane direction. The positive cross‐plane Seebeck is attributed to the lightly p‐doped nature of MAPbBr_3_. Negative in‐plane Seebeck is due to the negatively charged defects on the crystal surface which hinder the hole transport. To prove this hypothesis, an interfacial layer was coated on the top of the crystal surface that resulted in a positive Seebeck along the in‐plane direction. Xu et al. achieved a low *κ* and high Seebeck coefficient for centimeter‐sized MAPbI_3_ single crystal.^[^ ^134^ ^]^ For the temperature range 298 to 425 K, *κ* ranged from 0.3 to 0.42 Wm^−1^ K^−1^ while the Seebeck coefficient remained at 920 µV K^−1^ from 297 to 330 K before increasing to 1693 µV K^−1^ at 351 K. Similar to the *κ* measurements, a majority of the studies on thermoelectrics uses single crystalline halide perovskites.

**Figure 10:**
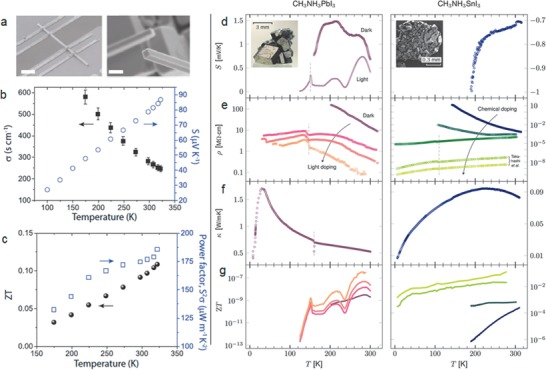
Individual AIHP NW is suspended between two membranes. The transport measurement direction is along the growth direction of the NWs. a) SEM image of the individual AIHP NW. Experimental data of thermoelectric properties in CsSnI_3_. b) Temperature‐dependent electrical conductivity (*σ*, black squares) and Seebeck coefficient (S, blue circles) of a single CsSnI_3_ NW. The positive sign of S indicates p‐type behavior, and CsSnI_3_ exhibits a relatively high electrical conductivity despite its ultralow *κ*. c) Figure of merit *ZT* (black dots) and power factor (blue squares) of single CsSnI_3_ NW. Power factor and *ZT* of as‐synthesized CsSnI_3_ before any attempts at optimization are 186 µW·m^−1^·K^−2^ and 0.11 at 320 K, respectively. Reproduced with permission.^[^ ^88^ ^]^ Copyright 2017, National Academy of Sciences. d–g) From top to bottom: thermoelectric power, S, electrical resistivity, *ρ*, thermal conductivity, *κ*, and figure of merit, *ZT*, of MAPbI_3_ (left panel) and MASnI_3_ (right panel) for light (photoelectron) and impurity doping. The three curves of MAPbI_3_, red, orange, and yellow correspond to light intensities of 80, 165, and 220 mW cm^−2^. The vertical dashed lines denote a structural phase transition observed in both MAPbI_3_ and MASnI_3_. Left inset: Optical image of a MAPbI_3_ crystal. Right inset: Scanning electron microscope image of a MASnI_3_ crystallite. Reproduced with permission.^[^ ^89^ ^]^ Copyright 2015, American Chemical Society.

In addition to hybrid perovskites, all‐inorganic halide perovskites are also investigated for thermoelectric applications. Guo et al. investigated the thermoelectric properties of CsPbI_3_ and CsSnI_3_ using a combination of first‐principles calculations and semiclassical Boltzmann transport theory.^[^ ^135^ ^]^ Low *κ_l_* of 0.54 and 0.25 Wm^−1^ K^−1^ at room temperature were computed for CsSnI_3_ and CsPbI_3_, respectively. *ZT* values at 1000 K for CsPbI_3_ and CsSnI_3_ are in the range of 0.4–0.65 for carrier concentrations in the order of 10^19^ cm^−3^. In an experimental work, Yang et al. reported ultralow *κ_l_* for single crystalline all‐inorganic halide perovskite nanowires (AIHP NW) of CsPbI_3_, CsPbBr_3_, and CsSnI_3_, close to the theoretical predictions.^[^ ^88^ ^]^ The *κ_l_* values of 0.45, 0.42, and 0.38 Wm^−1^ K^−1^ were measured for CsPbI_3_, CsPbBr_3_ and CsSnI_3_, respectively. Cluster rattling mechanism was attributed as the origin for the ultralow *κ* in these nanowires. Among these, CsSnI_3_ demonstrated a good electrical conductivity of 282 S cm^−1^ and a total *κ* of 0.57 Wm^−1^ K^−1^ at 300 K. A positive Seebeck value of 79 µV K^−1^ was obtained, resulting in a *ZT* of 0.11 at 320 K (**Figure** **10**a–c).

### 5.1 Effect of Self‐Doping, Intentional Doping, and Photodoping on Thermoelectric Performance

Point defects are responsible for self‐doping in hybrid perovskites and can be controlled, to a large extent, by manipulating the precursor ratios during growth. p‐ and n‐type self‐doping can be achieved by forming MAI‐rich and PbI_2_‐rich MAPbI_3_, respectively.^[^ ^136^, ^137^ ^]^ In one work, it was found that varying the concentration of chloride ions between pure MAPbI_3_ and mixed MAPbI*_x_*Cl_3−_ *_x_* can tune the carrier from p‐ to n‐type and consequently, the Seebeck coefficient.^[^ ^138^ ^]^ Apart from self‐doping, intentional doping of the B‐site metal cation in halide perovskites has been reported though their implications on thermal transport and thermoelectrics remain to be studied.^[^ ^139^, ^140^, ^141^ ^]^ In this section, we highlight some recent experimental and theoretical works on optimizing the performance of halide perovskite thermoelectrics by changing their carrier concentration through various doping strategies.

Shuai and coworkers using first‐principles calculations and Boltzmann transport equation predicted that tetragonal phase MAPbI_3_ has a superior thermoelectric performance than the cubic phase with the highest *ZT* occurring at the phase transition temperature of 330 K.^[^ ^142^ ^]^ For the tetragonal phase, the room temperature *ZT* value can be optimized to reach unity using a carrier concentration in the order of 10^18^ cm^−3^. Hole‐doped MAPbI_3_ was found to perform better than electron‐doped ones and can be achieved by engineering organic cation vacancies in MAPbI_3_. In contrast, DFT calculations from another work suggested that electron‐doped hybrid perovskites (MAPbI_3_, MASnI_3_, FAPbI_3_, and FASnI_3_) will perform better than hole‐doped ones.^[^ ^143^ ^]^ These discrepancies can be attributed to the use of different models and assumptions. In another theoretical work, MAPbI_3_ and MASnI_3_ based thermoelectrics with a carrier concentration of 10^18^ cm^−3^ could reach *ZT* values between 1 and 2 depending on the resulting *κ_l_*,^[^ ^27^ ^]^ showing good potential for solar thermoelectric applications.

Intentional doping strategies like those commonly used in organic and other material systems can be viable options to enhance the electrical conductivity of halide perovskites. Mettan et al. experimentally photo‐doped MAPbI_3_ single crystal and chemically doped MASnI_3_ polycrystal pellets for thermoelectric applications^[^ ^89^ ^]^ (Figure 10d–g). For MAPbI_3_, its electrical resistivity decreased by more than two orders of magnitude under illumination due to photoexcited carriers. As a result, its positive Seebeck coefficient dropped from 0.82 to 0.54 mV K^−1^ at 295 K and had a non‐monotonic temperature‐dependent behavior. In terms of its *ZT* value, it exhibited a low *ZT* of 10^−9^ in the dark that increased to around 10^−6^ under illumination (220 mW cm^−2^). In the case of MASnI_3_ pellets, illumination had no effect on its resistivity, which was measured to be lower than that of MAPbI_3_. A negative Seebeck coefficient of 0.72 mV K^−1^ at 295 K for MASnI_3_ was recorded in this work, resulting in a *ZT* value up to 0.13. Special doping strategies need to be designed to significantly improve the electrical conductivity of halide perovskites for thermoelectric applications.

In an interesting work, it was found that the positive Seebeck coefficient of MAPbI_3_ drops drastically at higher temperatures due to the tetragonal‐cubic phase transition that distorts the PbI_6_ octahedra.^[^ ^144^ ^]^ Surprisingly, under illumination and heating, the absolute magnitude of this Seebeck coefficient increased and changed sign (26 µV K^−1^ to −17 mV K^−1^). It was proposed that the holes are the majority carrier in the dark while the electrons become important under illumination. Furthermore, photoexcitation can increase the surface charge accumulation which serves as an additional driving force for the carriers to diffuse from the hot side to the cold side.

**Figure 11:**
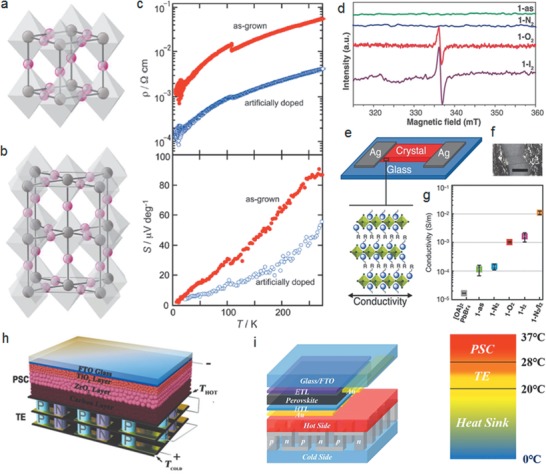
Crystal structure of MASnI_3_ at a) 295 K and b) 140 K. Sn is represented by the gray spheres and I by the pink spheres. MA cations are disordered and are not included. c) Change in the electrical resistivity and thermoelectric power caused by intentional doping. Reproduced with permission.^[^ ^145^ ^]^ Copyright 2011, Royal Society of Chemistry. d) X‐band electron paramagnetic resonance (EPR) of solid crystalline samples of 1‐as, 1‐N_2_, 1‐O_2_, and 1‐I_2_ at 77 K. e) The experimental setup used for single crystal electrical conductivity measurements and representation of the layered hybrid perovskites orientation showing the direction of conductivity measurements. f) Microphotograph showing a device made with a single crystal of 1‐ as; scale bar: 4 mm. g) Averaged values and standard uncertainties for in‐plane conductivity of [OA]_2_PbBr_4_, 1‐as, 1‐N_2_, 1‐O_2_, 1‐I_2_, and 1‐N_2_/I_2_ at 423 K. Reproduced with permission.^[^ ^146^ ^]^ Copyright 2018, Wiley‐VCH. h) Device architecture of perovskite photovoltaic‐Thermoelectric (PV‐TE) hybrid device with carbon top electrode. Reproduced with permission.^[^ ^147^ ^]^ Copyright 2018, Wiley‐VCH. i) Schematic device architecture of PV‐TE hybrid device with gold top electrode. Also shown is the simulated heat variation in the vertical direction of the PV‐TE hybrid system. Reproduced with permission.^[^ ^148^ ^]^ Copyright 2019, Elsevier.

Sn‐based hybrid perovskites exhibit higher electrical conductivity than their Pb counterparts but suffer from stability issues. In one work, MASnI_3_ was intentionally doped by replacing a portion of the SnI_2_ with SnI_4_ when making the growth solution, leading to one order of magnitude increase in its electrical conductivity (**Figure** **11**a–c).^[^ ^145^ ^]^ On the other hand, its positive Seebeck values and its trend with temperature decreased as a result of doping. The resulting concentration of holes was estimated to be in the order of 10^23^ cm^−3^, corresponding to a doping level of 0.003%. In contrast, Hao et al. observed a negative Seebeck of 60 µV K^−1^ at room temperature for MASnI_3_ with a corresponding carrier concentration of 10^14^ cm^−3^_._^[^ ^55^ ^]^ Different carrier concentration and sign of Seebeck for MASnI_3_ in these reports can be attributed to the impurities present in the precursors, defects generated during growth, and nature of impurity levels in the bandgap.^[^ ^55^, ^89^ ^]^ In another early work,^[^ ^149^ ^]^ a high negative Seebeck coefficient of ≈2.3 mV K^−1^ at 300 K was measured for Pb‐Sn mixed cation perovskite (MAPb_0.5_Sn_0.5_I_3_). More recently, Bi‐doped MAPbI_3_ film was explored for thermoelectric application.^[^ ^150^ ^]^ It was found that the Bi‐doping enhanced the thermal stability of the perovskite films. In addition, a simultaneous increase in the electrical conductivity and Seebeck coefficient (8 to 55 µV K^−1^) were observed, resulting in an enhanced power factor for doped films. Other intentional doping schemes for halide perovskites have been reported, but most focused on reducing the Pb content in the perovskite structure, improving the stability in case of Sn perovskites or enhancing the optoelectronic properties. There is a need for a more systematic investigation into the doping of halide perovskites to improve thermoelectric performance along with stability, an effort which seems to be gathering steam.

In a recent work, Fenwick et al. designed a strategy that combines self‐doping and chlorine doping of CsSnI_3_ to achieve a ZT value of 0.14 at 345 K for the resulting CsSnI_3−_ *_x_*Cl*_x_*. This is among the highest experimental *ZT* values reported for an all‐inorganic halide perovskite.^[^ ^127^ ^]^ It was observed that a thin chlorine rich surface acts as a free carrier source while protecting the bulk from oxidation. The self‐doping arising from the oxidation of Sn (ΙΙ) to Sn (ΙV) was tuned by controlled air exposure. The electrical conductivity and *κ* increased while the Seebeck decreased as a function of air exposure time with best *ZT* achieved after 6 min. *κ_l_* and Lorentz number were found to be 0.38 Wm^−1^ K^−1^ and 2.40 × 10^−8^ WΩ K^−2^ at room temperature, respectively. Compared to this work, two previous reports on CsSnI_3_ by Yang et al. and Saini et al. reported comparable *ZT* values of 0.11 (320 K) and 0.13 (300 K), respectively.^[^ ^88^, ^90^ ^]^ The CsSnI_3_ single crystalline nanowire in case of Yang et al. had a significantly higher electrical and thermal conductivity compared to thin film samples of Fenwick et al. and Saini et al.

**Table** **1** is a summary of some representative works on the state‐of‐the‐art halide perovskites with their *κ* and thermoelectric performance. Clearly, a variety of *κ* measurement techniques have been employed for halide perovskites, and almost all of them conclude the ultralow nature of *κ*. On the other hand, only a few works exist for studying the other thermoelectric parameters such as the Seebeck coefficient.

**Table 1:** Some representative works on halide perovskite *κ* and thermoelectric performance

Compound	*κ* measurement technique	*κ* [Wm^−1^ K^−1^]	S [µV K^−1^]	*ZT*	Ref.
MAPbI_3_	Steady‐state method	0.5 SC 0.3 PC			[20]
MAPbI_3_ (Theory)	MD	0.59 tetragonal 1.8 (330 K) pseudocubic			[71]
MAPbI_3_	Time‐domain thermal reflectance	11.2			[78]
MAPbI_3_ (Theory)	MD	<1 for all phases			[32]
MAPbI_3_	Laser flash	0.30–0.42 (298–425 K) SC	920 1693 (351 K)		[134]
MAPbI_3_	3*ω*‐scanning near‐field thermal microscopy	0.34 SC 0.33 PC			[87]
MAPbI_3_	Frequency domain thermoreflectance	0.34 SC			[86]
MAPbI_3_	Microbridge device	0.22 SC‐NW			[85]
MAPbI_3_	Laser flash	0.30 SC			[84]
MAPbI_3_	Micro‐photoluminescence	0.14 SC			[80]
MAPbI_3_	3*ω* technique	0.32 PC	−6500 5500		[82]
MAPbI_3_			820 (Dark) 540 (Light)	10^‐9^ (Dark) 10^‐6^ (Light)	[89]
MAPbI_3_			26 (Dark) −17000 (Light)		[144]
MAPbI_3_	Laser flash	0.38 (300–450 K) PC	−1350		[119]
MAPbI_3_	Laser flash	0.36 PC			[93]
MAPbI_3_	3*ω* technique	0.31–0.59 PC			[79]
MAPbBr_3_	3*ω*‐scanning near‐field thermal microscopy	0.44 SC 0.39 PC			[87]
MAPbBr_3_	Frequency domain thermoreflectance	0.51 SC			[86]
MAPbBr_3_	Microbridge device	0.32 SC–NW			[85]
MAPbBr_3_	Laser flash	0.37 SC			[84]
MAPbCl_3_	3*ω*‐scanning near‐field thermal microscopy	0.50 SC 0.50 PC			[87]
MAPbCl_3_	Frequency domain thermoreflectance	0.73 SC			[86]
MAPbCl_3_	Laser flash	0.52 SC			[84]
MASnI_3_	Steady‐state method	0.09 PC	−720	0.13	[89]
MASnI_3_			90		[145]
MASnI_3_			−60		[55]
(MA)_3_Bi_2_I_9_	Laser flash	0.21 (300–450 K) PC	2600		[119]
(MA)_3_Bi_2_I_9_	Laser flash	0.23 PC			[93]
FAPbBr_3_	Frequency domain thermoreflectance	0.49 SC			[86]
CsPbI_3_	NW thermometry technique	0.45 SC‐NW (*κ_l_*)			[88]
CsPbBr_3_	NW thermometry technique	0.42 SC‐NW (*κ_l_*)			[88]
CsPbBr_3_	Frequency domain thermoreflectance	0.46 SC			[86]
CsPbBr_3_	Microbridge device	0.36 SC‐NW			[85]
CsPbBr_3_	3*ω*‐scanning near‐field thermal microscopy	0.43 PC			[81]
CsPb_2_Br_5_	3*ω*‐scanning near‐field thermal microscopy	0.33 PC			[81]
CsPbCl_3_	3*ω*‐scanning near‐field thermal microscopy	0.49 PC			[94]
Cs_4_PbCl_6_	3*ω*‐scanning near‐field thermal microscopy	0.3 PC			[94]
CsSnI_3_	NW thermometry technique	0.57 SC‐NW 0.38 SC‐NW (*κ_l_*)	79	0.11 (320 K)	[88]
CsSnI_3_	3*ω* technique	0.36 PC	115	0.13	[90]
CsSnI_3_ Y_2_O_3_/CsSnI_3_	3*ω* technique	0.74 PC 0.30 PC	106 56	0.05 0.11	[91]
CsSnI_3−__*x*_Cl*_x_*	3*ω* technique	0.38–0.47 PC	103–144	0.14 (345 K)	[127]

SC, single crystal; NW, nanowire; PC, polycrystal; *κ*_l_, lattice thermal conductivity; MD, molecular dynamics. Blanks indicate property is not measured or calculated. Mentioned values are for room temperature unless indicated otherwise.

### 5.2 Challenges in Halide Perovskite Thermoelectrics

While current theoretical works predict halide perovskites as a promising material for thermoelectrics,^[^ ^131^, ^151^, ^152^ ^]^ it has been experimentally challenging to achieve decent performance. The low electrical conductivity of the relatively stable Pb‐based halide perovskites is a major hurdle for thermoelectrics which needs significant efforts to overcome. Although Sn‐based perovskites exhibit reasonable electrical conductivity, their instability limits their performance. Their long‐term stability in the presence of light, heat, moisture, and air is critical in ensuring their adaptation into functional commercial devices. On this front, the stability of halide perovskites is slowly improving and, in particular, several successful efforts to promote the longevity of the unstable Sn‐based perovskites have been reported for solar cells.^[^ ^153^, ^154^, ^155^, ^156^ ^]^ As Sn‐based perovskites have showed encouraging thermoelectric performance at lower temperatures, they can be targeted for room‐temperature applications. This improved stability can, perhaps, attract more efforts for the perovskite thermoelectrics which currently is limited to a few studies. Other than Pb‐ and Sn‐based hybrid perovskites, more investigations can be done on the relatively more stable all‐inorganic halide perovskites and double perovskites that can sustain higher operating temperatures. In contrast to solar cells, the smaller number of interfaces in thermoelectric device will help in improving the stability. Also encapsulation strategies developed for perovskite solar cells can be employed to enhance the stability of perovskite thermoelectrics.

While self‐doping is straightforward and capable of producing p‐ and n‐type perovskites, it may not increase the electrical conductivity to the level required for decent *ZT* values in Pb‐based perovskites.^[^ ^82^ ^]^ So far, attempts made to dope halide perovskites with different metal cations focused on optimizing their optoelectronic performances. The effects of doping on the thermal transport and thermoelectric properties of halide perovskites remain largely unexplored although the general trend can be inferred from other materials. Facile and effective doping strategy needs to be designed to improve the electrical conductivity of halide perovskites while preserving the structural integrity and stability without adversely affecting its thermal conductivity and Seebeck coefficient. Approaches like surface doping and applicability of advanced doping concept such as hyper‐doping need to be explored for improving the transport properties.^[^ ^157^, ^158^, ^159^ ^]^

The bandgap, low defect density, ultralow self‐doping of halide perovskites are attractive features for solar cell applications but not necessarily are favorable for thermoelectrics. In particular, the low carrier density limits the electrical conductivity of Pb‐based perovskites. Indeed, there are ways to tune the perovskites if the optoelectronic restriction is lifted. For instance, layered halide perovskites offer the possibility of intercalating conducting species between the spaces which has potential to improve the performance. This technique is known to improve the performance in layered inorganic thermoelectric materials.^[^ ^160^ ^]^ Also, new halide perovskites with favorable properties need to be designed for developing this class of materials for thermoelectrics.

In a recent work, a doping approach involving oxygen and iodine resulted in the bandgap reduction of a 2D perovskite and giant increase in the electrical conductivity up to three orders of magnitude.^[^ ^146^ ^]^ These 2D perovskites with polydiacetylenes (Figure 11d–g) in their structure were heated in different environments such as N_2_, air, and I_2_ resulting in electrical conductivity enhancement due to partial oxidation. This approach might be interesting to explore further on the condition that other properties are not adversely affected. Another approach of enhancing electrical conductivity can be through forming composites of halide perovskites with high mobility materials like in the case of organic thermoelectrics.^[^ ^161^ ^]^ Also, mixture of 3D/2D perovskites and alloyed B‐site perovskites can be explored to achieve improved stability.^[^ ^162^, ^163^ ^]^

## 6 Conclusions and Outlook

In this report, we highlighted the recent progress on thermal transport and phonon behaviors in halide perovskites as well as its nascent thermoelectric research. Both the potentials and challenges of halide perovskites as thermoelectric materials were analyzed. Halide perovskite offers interesting possibilities for studying uncommon phonon physics and creating efficient energy‐conversion devices like solar cells and thermoelectrics. Its application in the solar energy conversion domain has been a hot topic for the past decade with many advances since made. Research in thermoelectrics for this class of materials is, however, scarce and significant challenges need to be overcome before appreciable *ZT* can be realized. Their inherent ultralow *κ* and high Seebeck coefficient at room temperature are promising, but their low electrical conductivity has hindered the progress of halide perovskite thermoelectrics. We envision future advances would come from material optimization, new functionality, and device engineering for stability and performance enhancement in halide perovskite thermoelectrics.

Careful analysis of the ultralow nature of the *κ* value is needed as it directly affects the performance of perovskite‐based electronic devices like the thermoelectric *ZT* value. This effort has been progressing in the recent years. While current consensus holds that halide perovskites exhibit ultralow *κ*, there are some discrepancies in the literature pertaining to the *κ* values as well as its temperature‐dependent trend. These discrepancies are currently attributed, to some extent, to the different measurement techniques and conditions as well as the sample quality. Perovskite samples synthesized by different techniques can also lead to dissimilar electronic defects and charge transport characteristics resulting in contrasting results in different studies.^[^ ^164^ ^]^ The dependence of *κ* on the structural phase, dimensionality, and chemical compositions is getting clearer but still requires in‐depth experimental and theoretical investigations to tease out more details from this class of complex materials. Recent work on analyzing measurements of temperature‐dependent thermal diffusivity and specific heat during phase transitions in transient‐based measurement techniques may be applied to this class of materials to elucidate current uncertainties in their temperature‐dependent trends.^[^ ^165^, ^166^ ^]^

The effects and contributions from the intrinsic soft phonon modes on the thermal transport of a material is an important but largely unanswered question. How readily do a few soft phonon branches affect the overall thermal transport in any material? This class of material might be a good model for studying this question. In addition, effects on *κ* from the stress induced by their inherent large thermal expansion need further investigations for real world implementation. Mitigating schemes are also needed to release these stress in devices during actual operation. Apart from thermoelectrics, the nature of the *κ* trend is also crucial in the thermal management of functional devices. While ultralow *κ* of halide perovskites is a boon for thermoelectrics, it can pose serious implications for heat dissipation in solar cells and optoelectronic devices resulting in thermal stability issues. The actual temperature of a solar cell under operation can be significantly higher than the ambient temperature depending on the thermal properties of the active and charge transporting layers.^[^ ^167^ ^]^ Thermal endurance tests and proper thermal management strategies, be it new or adapted from existing thermal management systems,^[^ ^168^, ^169^ ^]^ need to be designed before perovskite‐based optoelectronic devices can be put into practical use. In addition, thermal conductivity‐dependent properties like thermo‐optic coefficients and hot‐carrier cooling are recently found to affect the energy‐conversion characteristics in these halide perovskites. The inherent ultralow *κ* created a giant thermo‐optic response in MAPbCl_3_ that enabled phase modulation of visible light.^[^ ^170^ ^]^ Slow cooling of energetic carriers due to the ultralow *κ* can potentially enhance the performance of perovskite‐based hot‐carrier solar cells.^[^ ^171^ ^]^

New innovative device designs and approaches should be developed to achieve maximum power output. As discussed in the previous section, photodoping of halide perovskites has shown some promising results though further optimization is imperative to improve their performance. In an interesting work, a photovoltaic‐thermoelectric (PV‐TE) hybrid device (Figure 11h) was fabricated to harvest both light and thermal energy to generate electricity.^[^ ^147^ ^]^ Consequently, the hybrid device generated an open‐circuit voltage of 1.29 V and a maximum point power output of 20.3% for 100 mW cm^−2^ AM 1.5 G illumination. In another recent work, an even higher average efficiency of 23.2% was achieved in PV‐TE hybrid device (Figure 11i) with MAPb(I_0.95_Br_0.05_)_3_ perovskite.^[^ ^148^ ^]^ A reasonable halide perovskite thermoelectrics device holds the key to future perovskite solar cell–thermoelectric hybrid devices. Instead of harvesting light and heat energy separately, light can be totally converted into heat before harvesting based on the concept of photothermoelectrics.^[^ ^172^ ^]^

Creating perovskites with new structures, composition, and functionalities should be explored to find the optimal performing materials for various operating conditions. In this regard, machine learning can be advantageous as thousands of perovskite compositions can be screened with high speed, saving experimental costs.^[^ ^173^, ^174^ ^]^ Apart from traditional hybrid and all‐inorganic halide perovskites, there are several other perovskite systems such as Pb‐ and Sn‐free halide perovskites and double perovskites which have received limited attention for thermoelectric applications. Recent theoretical calculations predict a high Seebeck coefficient for double perovskites Cs_2_AgBiX_6_ (X = Cl, Br).^[^ ^175^ ^]^ Interestingly, these double perovskites have higher Seebeck coefficient values than traditional thermoelectric material, Bi_2_Te_3_ under similar operating conditions. Furthermore, to the best of our knowledge, low dimensional perovskites such as 2D perovskites have yet to be explored for thermoelectrics. Recently, ultralow *κ* was observed for zero‐dimensional (MA)_3_Bi_2_I_9_ perovskite.^[^ ^119^ ^]^ Zero‐dimensional hybrids exhibit constrained charge and thermal transport.^[^ ^64^ ^]^ These lower dimensional materials can be explored as low‐temperature thermal insulation materials and microsensors expanding the functionality of halide perovskites.

Finally, theoretically predicted and experimentally observed emergent properties^[^ ^70^, ^111^, ^117^, ^176^ ^]^ such as strong phonon anharmonicity, lattice instability, symmetry breaking, and hot‐phonon bottleneck in halide perovskites need further investigations and understanding for modulating the thermal and related properties in future perovskite materials. Also, the broader implications of issues like ion migration and stability for thermoelectric performance need to be evaluated.^[^ ^177^, ^178^, ^179^ ^]^ More efforts are needed to identify potential halide perovskites for thermoelectrics as well as to develop novel strategies for improving their electrical conductivity. A multidisciplinary research effort involving theoretical and experimental approaches will be needed to overcome challenges for realizing high‐performance halide perovskite‐based thermoelectrics.

## Conflict of Interest

The authors declare no conflict of interest.
